# Pyogenic liver abscess complicated with endogenous endophthalmitis caused by *Klebsiella pneumoniae*: A case report and Literature Review

**DOI:** 10.1002/iid3.943

**Published:** 2023-07-27

**Authors:** Yunjiang Chen, Yanchun Gong, Bei Song, Yueling Du, Kaiyu Cai

**Affiliations:** ^1^ Department of General Practice Ruijin Hospital, Shanghai Jiao Tong University School of Medicine Shanghai People's Republic of China

**Keywords:** case report, endogenous endophthalmitis, *K. pneumoniae*, pyogenic liver abscess

## Abstract

**Objective:**

Pyogenic liver abscess (PLA) is a common surgical infectious disease caused by various pathogens. *Klebsiella pneumoniae* is a relatively recent cause, often affecting patients with low immunity. Endogenous endophthalmitis (EE), a rare and serious complication of PLA, may appear with eye symptoms before PLA. By reviewing a case of *Klebsiella pneumoniae*‐induced PLA complicated with EE, we want to summarize the information about the characteristics, causes, and complications of PLA based on the literature review.

**Methods:**

This case report describes a 37‐year‐old male who had fever high to 39°C for 10 days experienced blurred vision followed by nonlight perception vision. He reported a history of diabetes irregularly taking oral medications and insulin therapy. Imaging examination found a large low‐density area in the right lobe of the liver with an unclear border and vague surrounding fat gap. The blood culture was not positive. The culture of the drainage fluid from the liver puncture showed *Klebsiella* pneumonia. Blood and liver puncture drainage fluid were sent for microbial high‐throughput gene detection with next‐generation sequencing technology (NGS), which confirmed the diagnosis of *Klebsiella pneumoniae*‐induced PLA complicated with EE.

**Results:**

The patient's surgical incision had healed well at discharge, and he could feel light at his left eye. But the patient was lost to follow‐up since the third month after discharge.

**Conclusion:**

By reviewing this case and summarize the information about the characteristics, causes, and complications of PLA based on the literature review, we concluded that it is necessary to promptly perform liver puncture drainage and empirically use antibiotics for patients with PLA, especially those with poor glycemic control, to avoid serious complications such as EE.

## Graphical abstract

It is important to promptly perform liver puncture drainage and empirically use antibiotics for patients with PLA, especially those with poor glycemic control, to avoid serious complications such as endogenous endophthalmitis.

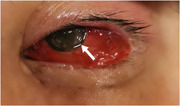

## 1 INTRODUCTION

Pyogenic liver abscess (PLA) is a common surgical infectious disease caused by various pathogens such as bacteria, fungi, and parasites. It can occur in various conditions such as biliary tract disease, colitis, pancreatitis, intravenous drug use, and trauma.^1^, ^2^

*Klebsiella pneumoniae* is a type of Gram‐negative bacteria that is commonly found in the human gut and respiratory tract. In addition to PLA, *K. pneumoniae* infections can also cause pneumonia, urinary tract infections, and other types of infections.^3^ *K. pneumoniae* is a commensal bacterium that lives in the nasal cavity and gastrointestinal tract and is frequently responsible for community‐acquired pneumonia or urinary tract infections. When it causes liver abscesses, we call them invasive liver abscesses. This invasive PLA is caused by *K. pneumoniae* strains expressing serotypes K1 and K2 as well as mucoid phenotype A regulator.^4^, ^5^ Because of underlying conditions such as diabetes and tumors, patients with this type of PLA frequently have low immunity.^1^, ^2^, ^6^, ^7^, ^8^ De Francesco described for the first time in Italy, a case of PLA caused by an ST‐23 hypervirulent *K. pneumoniae* (hvKp, a strain that can cause infections in relatively healthy people, often in community settings) strain and detected in an immunocompetent Chinese subject.^9^ The pathogenesis of *K. pneumoniae*‐induced PLA is not fully understood, but it is believed to involve bacterial invasion and destruction of liver cells, as well as the production of toxins and immune system activation.^10^

In addition to diabetes and tumors, other risk factors for *K. pneumoniae*‐induced PLA include alcoholism, chronic kidney disease, and recent invasive procedures such as endoscopy or surgery.^11^ Various virulence factors have been demonstrated to aid in the infectivity of *K. pneumoniae*. Capsule, lipopolysaccharide, adhesin, and siderophores are among the virulence factors found in carbapenem‐resistant *K. pneumoniae* (CRKP)/hvKp, resulting in a variety of immune responses and related phenotypes in hvKp strains.^12^

The presence of *K. pneumoniae‐*induced PLA is usually confirmed by imaging studies such as ultrasound or computed tomography (CT) scan, as well as blood cultures. They are useful tools for demonstrating a space‐occupying lesion and confirming the presence or absence of a liver abscess. CT scanning has a higher sensitivity (97% sensitive) for detecting liver abscess than ultrasound (85% sensitive).^13^

In patients with *K. pneumoniae*‐induced PLA, CT findings such as abscess location (i.e., left or both lobes) and involvement of adjacent vessels, in addition to ocular symptoms, should be considered for an early diagnosis of EE.^14^ Treatment typically involves a combination of antibiotics and drainage of the abscess if necessary. *K. pneumoniae* was still highly susceptible to ceftazidime and amikacin, and the multidrug‐resistant (MDR) *K. pneumoniae* isolates were not common in EE.^15^ Current suggestions are that liver abscesses less than 3 cm can be treated medically. In a large majority of patients, aspirations of liver abscesses are successful and result in resolution.^16^ Fine needle aspiration for culture is the gold standard for diagnosis of PLA. Blood cultures are an important adjunct to the diagnosis of pyogenic abscess and although their yield is usually lower than pus aspirate of PLA, It is recommended to perform a blood culture for any patient suspected of PLA on entry.^17^, ^18^

Endogenous endophthalmitis (EE) is a rare and serious complication of PLA and often appears with eye symptoms before PLA.^8^, ^19^, ^20^ Despite continued negative blood cultures, the patient's presentation may be consistent with invasive liver abscess syndrome, with associated EE qualifying as a systemic manifestation of disease.^21^ A meta‐analysis estimates the actual incidence of EE among PLA patients, where EE is reported in approximately one out of every 22 patients with *Klebsiella pneumoniae* pyogenic liver abscess. K1 capsular serotype infection was an independent risk factor.^22^ Here, we report a case of EE caused by *K. pneumoniae‐*induced PLA.

### 1.1 Case presentation

A 37‐year‐old male was admitted to the hospital 1 day after left eye surgery and more than 10 days of fever. He reported having a fever more than 10 days ago, accompanied by chills and shivering, with the highest body temperature being 39°C. He purchased fever‐reducing drugs from a pharmacy without seeking medical attention. Two days before admission, he began to experience blurred vision in the left eye, followed by nonlight perception vision. He went to Eye & Ent Hospital of Fudan University for emergency treatment and underwent left lens and vitreous body removal under local anesthesia. During the surgery, vancomycin and cefotaxime were injected, and silicone oil was injected into the eyeball. The surgery was successful, and a large area of retinal necrosis, large temporal hiatus in macular area, and extensive vascular occlusion were observed during the surgery. The bacterial smear of purulent secretion during surgery indicated Gram‐positive bacteria, and no fungi were found. The patient was immediately given Rocephin for anti‐infective treatment after surgery.

On the second day after surgery (September 29, 2020), he was admitted to Ruijin Hospital affiliated to Shanghai Jiao Tong University School of Medicine. His body temperature was 39.3°C, and he had no chills. He had a good appetite but poor sleep, normal bowel, and urine habits, and had lost 5 kg in weight in the past month.

### 1.2 Past medical history

The patient had a history of diabetes for more than 3 years, irregularly taking oral medications and insulin therapy. He denied a history of hypertension and chronic diseases. He had a history of hemorrhoids and had bleeding from hemorrhoids 1 week before the fever.

### 1.3 Physical examination

On examination, the patient had a body temperature of 39.3°C, R22 breaths/min, BP124/85 mmHg, HR96 beats/min, and the left eye was covered with gauze. No abnormalities were found in heart and lungs, and the abdomen was soft and flat. The liver was palpable one finger breadth below the ribs, with mild tenderness upon gentle pressure.

### 1.4 Diagnostic assessment

#### 1.4.1 Biochemical examination

Upon admission, a blood culture specimen was collected and yielded the following results: white blood cell count (WBC) 13.3 × 10^9^/L (normal range: 3.97–9.15 × 10^9^/L), with neutrophils accounting for 86.2%; erythrocyte sedimentation rate (ESR) 74 mm/h; C‐reactive protein (CRP) 276.1 mg/L; procalcitonin 1.06 ng/mL; serum 1, 3‐beta‐d‐glucan level (G tes) (−); endotoxin (−); 2‐h postprandial blood glucose 16.13 mmol/L; and glycated hemoglobin (HbA1c) 10.2%. The patient was started on antibiotics including Vancomycin 1.0 g q12h, Piperacillin‐Tazobactam 4.5 g q8h, and Metronidazole 0.5 g q12h after the blood culture specimen was collected.

#### 1.4.2 Imaging examination

**Figure 1:**
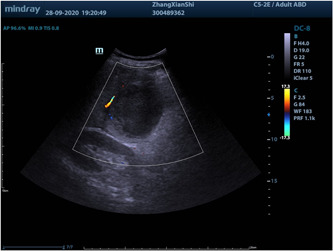
Mixed echogenicity with septation (white arrow) seen in the right lobe of the liver, measuring approximately 95 × 80 mm.

**Figure 2:**
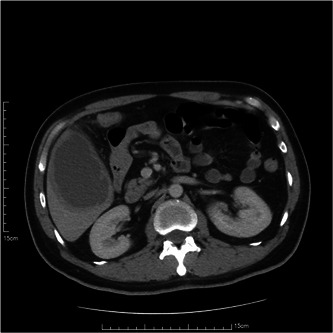
The delayed phases of contrast‐enhanced computed tomography show a low‐density lesion in the right lobe of the liver, measuring approximately 9.3 × 6.1 cm, with thickening of the cyst wall (white arrow).

**Figure 3:**
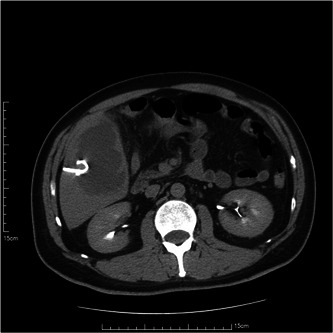
Liver puncture catheter placement for drainage of pus.

**Figure 4:**
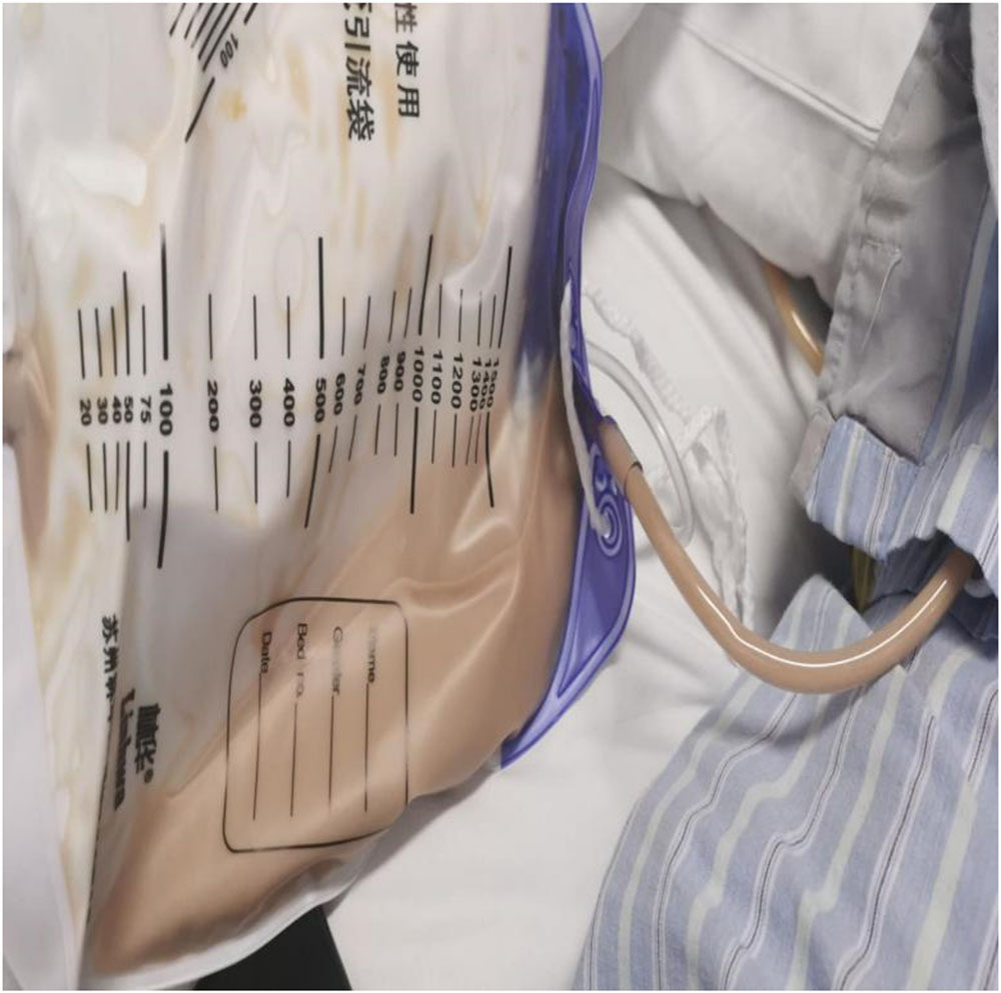
Liver puncture drainage resulted in coffee‐colored purulent material, and culture of the drainage fluid grew *Klebsiella pneumoniae*.

An abdominal ultrasound, which involved an abdominal ultrasound that specifically targeted the right posterior lobe of the liver, was conducted upon admission. and showed a mixed echoic area with a thick wall and internal partitions, about 95 × 80 mm in size, with an unclear border and slightly enhanced posterior echo signal, and a few blood flow signals were seen in the wall on color Doppler flow imaging (CDFI) (Figure 1). On the second day of admission, an upper abdominal contrast‐enhanced CT was performed and showed a large low‐density area in the right lobe of the liver with an unclear border, measuring 8.5 × 5.8 cm, and the surrounding fat gap was vague. The diagnosis was a PLA in the right lobe with slight exudative changes around it (Figure 2). A percutaneous liver puncture and drainage tube were placed under CT guidance (Figures 3 and 4).

**Figure 5:**
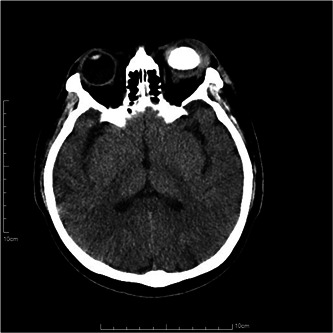
The swelling of subcutaneous tissue around the eye following removal of the lens and vitreous, and injection of silicone oil.

**Figure 6:**
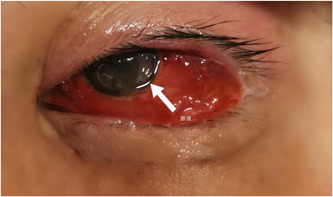
Postoperative local purulent discharge in the left eye, and bacterial smear from another hospital showed Gram‐positive bacteria.

A head CT scan was performed and showed increased density in the left eye and swelling of the subcutaneous tissue around the left eye (Figure 5). After consultation with the Infectious Disease Department, the patient's antibiotic treatment was adjusted to Vancomycin 1.0 g q12h, Meropenem 1.0 g q8h, and Metronidazole 0.5 g q12h. On the third day of admission, the patient had purulent discharge from the left eye under the cornea, and ophthalmology consultation recommended debridement and dressing change (Figure 6).

#### 1.4.3 Microbiology examination

The blood culture was not positive. The culture of the drainage fluid from the liver puncture showed *Klebsiella pneumonia*, and the results of the drug sensitivity test showed that it was not sensitive to ampicillin but moderately sensitive to vancomycin, cephalothin, and meropenem. Blood and liver puncture drainage fluid were sent for microbial high‐throughput gene detection, which confirmed the diagnosis of *K. pneumonia* induced‐PLA with EE.

### 1.5 Treatment and outcomes

The patient received antibiotic treatment and blood sugar control (insulin detemir 18 units subcutaneous injection once nightly, insulin aspart 6 units subcutaneous injection 15 min before each meal, metformin/sitagliptin 50/500 mg twice daily orally, and linagliptin 10 mg once daily orally) during a 2‐week hospital stay. Ten days into the hospitalization, the patient reported feeling better and requested to be transferred to a hospital near their residence for continued treatment of the infection. Upon discharge, the patient had been afebrile for 10 days and a follow‐up CT scan of the upper abdomen showed a significant reduction in the size of a large low‐density lesion in the right lobe of the liver, measuring approximately 4.6 × 2.5 cm. The patient's surgical incision had healed well, and he could feel light at his left eye. The patient was discharged and transferred to a local hospital for convenient nearby medical treatment on October 12, 2020. One month after transfer, the patient reported via telephone follow‐up that he had been discharged from the local hospital after recovery with light perception of left eye (no specific vision was reported at this follow‐up). We tried to contact the patient via telephone at the 3rd, 6th, and 12th months, but the patient was lost to follow‐up since the 3rd month after discharge.

## 2 DISCUSSION

EE is an ocular infection that occurs secondary to sepsis, with an incidence of 0.04%–0.5% in cases of bacteremia or fungemia.^23^ The incidence of PLA in EE patients has been reported to be 0.84%–6.9%,^24^ making it one of the most serious complications of PLA. PLA is a rare but potentially fatal infectious disease in Western populations, with an incidence of 20 cases per 100,000 hospitalized patients.^25^ Recent studies in China have shown that the bacteria causing PLA have shifted from *Escherichia coli* to *K. pneumoniae*, which now accounts for the majority of cases.^26^, ^27^ *K. pneumoniae* is the most common pathogenic bacteria, and most early cases of *K. pneumoniae*‐induced PLA with concomitant EE have been reported in Taiwan.^28^ A 12‐year retrospective study in Taiwan showed that *K. pneumoniae* was responsible for 55.8% of cases of EE.^6^ A survey of data from 1996 to 2015 in Taiwan found that there were 104 cases (120 eyes) of PLA with concomitant EE, with unilateral involvement accounting for the vast majority (84.6%), solitary PLA accounting for 74%, and right lobe abscess accounting for 60.6%.^24^ The incidence of diabetes in these cases was 68.3%, similar to other studies,^6^ and EE appeared before PLA in 52.1% of cases. Recent research from the Second Affiliated Hospital of Nanchang University showed that trauma accounted for 71% of cases of endophthalmitis, while ocular surgery accounted for 18% and EE accounted for 7.8%.^29^ Foreign data show that the incidence of EE in cases of endophthalmitis is 7.4% in India, 16.5% in Thailand, and 23.5% in the United States.^1^, ^30^, ^31^, ^32^

*K. pneumoniae* endophthalmitis is increasingly reported globally and is a primary cause of EE in Southeast Asian countries, where Gram‐negative bacteria are the primary causative agents. In contrast, Western countries report Gram‐positive bacteria and fungi as the most common pathogens. This type of endophthalmitis has a higher incidence among East Asian populations, particularly in patients with poorly controlled diabetes, with PLA being the most common source of infection.^24^, ^32^, ^33^, ^34^, ^35^

PLA caused by *K. pneumoniae* can occur through four pathways^36^: (1) the biliary tract pathway, which is usually associated with cholelithiasis, malignant tumors, or posthepaticojejunostomy stricture^37^; (2) the portal vein pathway, which mainly originates from abdominal infections such as appendicitis, diverticulitis, colorectal cancer, and inflammatory bowel disease^38^; (3) the hepatic artery pathway, which is usually caused by bacteremia from *Staphylococcus aureus* ^38^; and (4) direct infection from adjacent infected foci. Although some studies have suggested that *K. pneumoniae*‐induced PLA is seldom associated with liver and biliary diseases, previous liver surgery, abdominal trauma, or biliary or portal vein infections caused by malignant tumors,^39^, ^40^ mouse model experiments have demonstrated that different mutant strains of *K. pneumoniae* have varying invasive abilities through different genes. Type III mutants, for example, can penetrate the intestinal barrier and cause PLA.^41^

The patient in this case had a history of bleeding hemorrhoids 1 week before developing a fever. Intestinal colonization by both *K. pneumoniae* and Gram‐positive bacteria can lead to the formation of PLA in the right lobe through the blood supply pathway of the hemorrhoid plexus.^42^ Since intravenous drugs have limited ability to penetrate the blood–brain and blood–retinal barriers, an empiric treatment approach involving intravitreal injection of vancomycin and cefotaxime was taken during the patient's left eye vitrectomy and lens removal surgery to target Gram‐positive bacteria.^43^ However, subsequent bacterial smear of the eye discharge confirmed Gram‐positive bacteria, which was inconsistent with the result of the liver puncture drainage fluid culture and blood detection with next‐generation sequencing technology (NGS) that showed *K. pneumoniae*.^43^

The clearance of Gram‐positive bacteria by vancomycin could have contributed to the inconsistency with the results of liver puncture drainage fluid culture, which was taken after empirical antibiotic treatment for Gram‐positive bacteria.^24^ Although intravitreal steroid therapy has been shown to increase the chances of saving vision in EE patients, all patients suspected of infection receive empirical systemic antibiotic treatment before a PLA diagnosis is confirmed.^24^ Nonetheless, in this case, the patient had a good outcome without steroid therapy. Vancomycin inhibits the synthesis of the cell wall of Gram‐positive bacteria, rendering them unable to survive, and is therefore mostly ineffective against Gram‐negative bacteria.^2^ *K. pneumoniae* is a common Gram‐negative bacterium and is typically susceptible to carbapenems, gentamicin, and cefotaxime.

During the patient's surgery, vancomycin and cefotaxime were injected into the eye, and vancomycin combined with meropenem was administered after the operation. Following 2 weeks of antibiotic treatment, the patient no longer had a fever, and follow‐up CT scans showed a significant decrease in the PLA, indicating that the chosen antibiotic treatment was effective.

Antibiotics should be adjusted based on drainage fluid and/or blood culture results.^7^ According to the guidelines for intra‐abdominal infections by the Infectious Diseases Society of America (IDSA), the recommended duration of antimicrobial therapy for *K. pneumoniae‐*induced PLA is 4–6 weeks, with the duration of treatment being based on control of the primary focus.^44^ For the treatment of EE, surgical vitrectomy and empirical intravitreal injection of drugs are still the preferred options during the acute phase. However, there is still no consensus on the indications and timing of vitrectomy, and the protective effect of vitrectomy on vision and the eyeball remains controversial.^1^, ^43^, ^45^ In addition, the administration of dexamethasone via intravitreal injection has not been shown to have a significant correlation with preservation or good visual acuity in the eye.^34^

## 3 CONCLUSION

It is important to promptly perform liver puncture drainage and empirically use antibiotics for patients with PLA, especially those with poor glycemic control, to avoid serious complications such as EE.

## AUTHOR CONTRIBUTIONS

**Yunjiang Chen**: Conceptualization; data curation; investigation; methodology; writing—original draft; writing—review and editing. **Yanchun Gong**: Conceptualization; data curation; investigation; methodology; project administration; writing—original draft. **Bei Song**: Data curation; investigation; methodology; resources; writing—review and editing. **Yueling Du**: Investigation; methodology; project administration; supervision; writing—review and editing. **Kaiyu Cai**: Conceptualization; investigation; methodology; supervision; validation; writing—review & editing.

## ETHICS STATEMENT

The patient has signed a copy of written informed consent for the notice of this case research and publication.

## Data Availability

The related data can be accessed from the corresponding author.
